# Efficacy and safety of controlled‐release dinoprostone vaginal delivery system (PROPESS) in Japanese pregnant women requiring cervical ripening: Results from a multicenter, randomized, double‐blind, placebo‐controlled phase III study

**DOI:** 10.1111/jog.14472

**Published:** 2020-10-22

**Authors:** Hiroaki Itoh, Keisuke Ishii, Naoya Shigeta, Atsuo Itakura, Hiromi Hamada, Takeshi Nagamatsu, Tomohiko Ishida, Yasuaki Bungyoku, Ali Falahati, Miori Tomisaka, Mikiya Kitamura

**Affiliations:** ^1^ Department of Obstetrics and Gynecology Hamamatsu University School of Medicine Higashi‐ku Hamamatsu Japan; ^2^ Osaka Women's and Children's Hospital Izumi Japan; ^3^ Rinku General Medical Center Izumisanoshi Osaka Japan; ^4^ Juntendo University Bunkyo‐ku Tokyo Japan; ^5^ University of Tsukuba Tsukuba Ibaraki Japan; ^6^ The University of Tokyo Bunkyo‐ku Tokyo Japan; ^7^ Itabashi Chuo Medical Center Itabashi‐ku Tokyo Japan; ^8^ Ferring Pharmaceuticals Co. Ltd. Minato‐ku Tokyo Japan

**Keywords:** cervical ripening, dinoprostone, uterotonic drug, vaginal delivery, vaginal delivery system

## Abstract

**Aim:**

To evaluate the efficacy and safety of dinoprostone vaginal insert (PROPESS) in pregnant post‐term Japanese women requiring cervical ripening.

**Methods:**

This randomized, double‐blind, placebo‐controlled study included 114 pregnant Japanese women at term (41 weeks of gestation) requiring cervical ripening (baseline Bishop score (BS) ≤ 4). The primary end‐point was the proportion of subjects with successful cervical ripening defined as BS ≥ 7 or vaginal delivery in 12 h. The secondary end‐points were changes in BS, proportion of women with vaginal delivery, proportion of women receiving mechanical cervical ripening procedure and use of oxytocic drugs.

**Results:**

PROPESS administration for a maximum of 12 h showed significantly higher successful cervical ripening rate (47.4% vs 14.3%, respectively; treatment contrast [TC]: 33.1%; *P* = 0.0002). The median time from administration to vaginal delivery was significantly shorter in the PROPESS group than in the placebo group (26.18 h vs 33.02 h; OR 2.51; 95% CI [1.60–3.92]; *P* < 0.0001). In the PROPESS group, the dosage of uterotonic drugs, such as oxytocin, decreased, and the number of patients who used these drugs also decreased.

**Conclusion:**

PROPESS administration for a maximum of 12 h was an effective and well‐tolerated treatment for pregnant Japanese women post‐term requiring cervical ripening.

## Introduction

Cervical ripening in the last trimester of pregnancy is a very important process prior to the onset of delivery.^1^ During pregnancy, the cervix remains firm, long and closed to retain the fetus and prevent miscarriage and preterm delivery, but it gradually starts to soften late in the first step of labor, and ripens as effacement and dilation occur. Insufficient cervical ripening is an obstacle of vaginal delivery; alternatively, a cesarean section could be performed, but it exposes the fetus and mother to unnecessary risks. Therefore, cervical ripening intervention at the appropriate time is required for women who need induction of labor and have a cervix that has a Bishop score (BS) < 6 as we know that induction of labor with oxytocin has a high risk of failure when the BS is <6.

BS is generally used worldwide as an end‐point of cervical ripening. BS is a subjective cervix evaluation based on manual examination. In the diagnosis of failure of cervical ripening, BS ≤ 6 is considered to indicate failure of cervical ripening in many randomized studies, in accordance with the 2009 American College of Obstetricians and Gynecologists (ACOG) guidelines^2^ and Canadian guidelines.^3^ UK guidelines recommend transvaginal administration of a prostaglandin E2 (PGE2) product to induce labor regardless of BS or membrane rupture.^4^

In Japan, Guidelines for Obstetrical Practice in Japan: Japan Society of Obstetrics and Gynecology (JSOG) and Japan Association of Obstetricians and Gynecologists (JAOG) 2020 edition states the following: 'There are no uniform criteria for cervical ripening, but in general a BS ≤ 6 is often regarded as poor cervical ripening (CQ412)'.^5^ Therefore, insufficient cervical ripening is often defined as BS ≤ 6 overseas and in Japan.

Treatments for cervical ripening can generally be classified as mechanical or pharmacological therapy. PROPESS has been used around the world already and is recommended as the standard treatment in various guidelines worldwide.^2^, ^3^, ^4^, ^5^ Some studies showed the efficacy of PROPESS compared with placebo.^6^, ^7^, ^8^ Cervical ripening using PGE_2_ softens and dilates the cervical canal, successfully inducing labor and shortening the time to delivery.

In Japan, the only approved product for pharmacologically induced cervical ripening before the approval of PROPESS was an intravenous injectable formulation of sodium prasterone sulfate hydrate. Therefore, mechanical cervical ripening methods have been more commonly used. Controlled‐release dinoprostone vaginal insert (PROPESS Vaginal Delivery System 10 mg) is a drug that promotes cervical ripening in pregnant women. PROPESS is composed of a thin, flat, hydrogel polymer containing 10 mg of dinoprostone enclosed within a mesh‐knitted thread for retrieval. PROPESS is considered an alternative administration route owing to the following characteristics^9^: (i) PROPESS has a drug delivery system that releases the dinoprostone in a controlled and constant rate of approximately 0.3 mg/h over 12 h; (ii) its retrieval system enables easy and quick removal at the onset of labor or in the event of complications; and (iii) one administration is usually enough to produce satisfactory effects.

Thus, in the present phase 3 clinical study, we aimed to examine the comparative efficacy and safety of PROPESS versus placebo (an identical structure without dinoprostone) in pregnant women at ≥41 weeks 0 day to ≤41 weeks 6 day of gestation with BS ≤ 4. The study was preregistered n 'ClinicalTrials.gov' (the clinical trial number is NCT03067727).

## Methods

### Study design

The present study was a multicenter, placebo‐controlled, randomized, double‐blind study designed to evaluate the efficacy and safety of PROPESS in Japanese pregnant women at 41 weeks of gestation who required labor induction. Women who required cervix ripening (BS ≤ 4) before induction of labor were asked to participate considering previous cohort studies^6^, ^8^, ^10^, ^11^. The study was conducted in 19 sites in Japan from April 2017 to August 2018.

The study was approved by the institutional review board for each site and conducted in accordance with the Declaration of Helsinki and ethics guidelines for clinical research. All patients provided written informed consent before participating in the study.

### Patients

The eligibility criteria in this study were pregnant women with gestational age ≥ 41 weeks 0 day and ≤ 41 weeks 6 day, aged ≥20 years and requiring labor induction.

Subjects who signed an informed consent form were evaluated for eligibility during the Screening visit and/or Baseline visit. Demographics, body measurements, obstetric status, obstetric and medical/surgical history, physical examination of the mother, gynecological examination, BS, laboratory results, fetal monitoring (CTG) and vital signs were assessed.

The major inclusion criteria were as follows: (i) pregnant women at term ≥41 weeks 0 day and ≤ 41 weeks 6 day at the baseline visit; (ii) women aged ≥20 at the screening visit; (iii) candidates for pharmacological labor induction; (iv) patients with insufficient cervical ripening, defined as baseline BS ≤ 4 at baseline visit; (v) women with singleton pregnancy with live fetus in the vertex presentation; and (vi) patients with parity ≤ 3 (parity is defined as one or more live births or stillbirths after 22 weeks 0 day of gestation).

The major exclusion criteria were as follows: (i) women in labor; (ii) women with uterine or cervical scars, including scars from previous cesarean section and previous cone biopsy of the cervix and loop electrosurgical excision procedure; (iii) women with uterine abnormality (e.g. bicornate uterus); and (iv) women administered oxytocin, any cervical ripening or labor‐inducing agents (including mechanical methods) or a tocolytic drug within 7 days prior to Investigational Medicinal Product (IMP) administration.

### Study treatment and procedure

Eligible subjects were randomly assigned to either the PROPESS or placebo group, with stratification factors being obstetric history (nulliparous and multiparous) and BS (≤2 and ≥3); each subject received IMP for up to 12 h and were followed up after delivery until discharge. The randomization number was allocated by the electric Case Report Form (eCRF) in the order in which the subjects were being randomized into the trial.

To confirm eligibility, demographics, body measurements, obstetric status, obstetric and medical/surgical history, physical examination of the mother, gynecological examination, BS, laboratory results, fetal monitoring (CTG) and vital signs were assessed. CTG monitoring was performed for at least 20 min prior to IMP insertion to confirm no evidence of nonreassuring fetal heart rate (FHR) pattern and no uterine contractile pattern indicative of labor as a part of eligibility verification. CTG was continued during IMP administration to ensure the absence of fetal dysfunction and uterine contractions suggestive of labor pain.

Vital signs were monitored every hour throughout the 12‐h period after IMP insertion. BS was evaluated at 3, 6, 9 and 12 h after insertion. If 12 h had elapsed after insertion, the IMP was removed regardless of the presence or absence of cervical ripening. In addition, the IMP was removed immediately in the event of start of active labor, membrane rupture or amniotomy, an adverse event during labor requiring removal of IMP or the (sub)investigator deemed it necessary to stop the study during the 12‐h administration period.

If induction or stimulation of labor was necessary, a uterotonic agent was used at least 60 min after IMP removal, and if a mechanical cervical ripening procedure was required, the procedure was performed at least 60 min after IMP removal.

During the second stage of labor, the following parameters were recorded: date and time of labor onset; date and time of start of active labor; and date, time and method of membrane rupture. At birth, date and time of fetal extraction, delivery method, newborn body weight, Apgar score at 1 and 5 min and newborn physical findings were evaluated, and umbilical artery blood gas analysis was performed. In addition, neonatal intensive care unit (NICU) entry and exit data, as well as the reason, were recorded, if applicable.

The mother's physical findings and vital signs were evaluated for 48 h after delivery, and findings of vaginal examination, physical findings of mothers and newborns, vital signs and laboratory findings were evaluated at discharge. In addition, NICU entry and exit data were recorded, if applicable.

### Study end‐points

The primary efficacy end‐point was the proportion of subjects with cervical ripening success within 12 h after vaginal insert administration, defined as either BS ≥ 7 or vaginal delivery within 12 h after insertion.

The secondary efficacy end‐points included proportion of nulliparous and multiparous subjects with cervical ripening success within 12 h after insertion; proportion of subjects with an increase in BS from baseline ≥3 at 12 h after insertion; proportion of subjects who underwent cesarean section during the first admission to hospital; proportion of subjects who used predelivery oxytocic drugs after the removal and dosing of predelivery oxytocic drugs; proportion of subjects who underwent mechanical cervical ripening procedure after PORPESS or placebo removal; time of mechanical cervical ripening procedure in subjects who underwent mechanical cervical ripening procedure after IMP removal; time to start of active labor after insertion during the first admission to hospital; and time to vaginal delivery, cesarean section and delivery by any delivery method after insertion during the first admission to hospital.

Safety and tolerability of PROPESS treatment were evaluated based on hematology, blood chemistry, urinalysis, vital signs, physical findings and vaginal examination; these were usually monitored in clinical research.^6^, ^8^, ^10^, ^11^ adverse event (AE) was coded to system organ class and preferred term in accordance with the latest Medical Dictionary for Regulatory Activities.

### Statistical analyses

Primary efficacy was assessed for the full analysis set (FAS), which consisted of all intention‐to‐treat (ITT) subjects who, after IMP insertion, had at least one nonmissing BS value or a vaginal delivery within 12 h.

The primary efficacy end‐point, that is, the proportion of subjects with cervical ripening success within 12 h of vaginal insert administration, was analyzed using Fisher's exact test at 12 h. The proportion and two‐sided 95% confidential interval (CI) for cervical ripening success within 12 h were presented. The proportion analyzed using Fisher's exact test was not adjusted.

Secondary efficiency was analyzed using Fisher's exact test in the proportion of nulliparous and multiparous subjects with cervical ripening success within 12 h, proportion of subjects delivering vaginally within 12 h and proportion of subjects with increased BS from baseline ≥3 at 12 h. The proportion of subjects delivering vaginally within the first admission to hospital, proportion of subjects who underwent cesarean section during the first admission to hospital and proportion of subjects with BS ≥ 7 at the onset of labor among those having onset of labor was analyzed using analysis of CO‐variance, with baseline BS as a covariate, as well as the treatment, nulliparous or multiparous and baseline BS (≤2, 3≤) as fixed effects. The proportion of subjects who used predelivery oxytocic drugs after the removal and proportion of subjects who underwent mechanical cervical ripening after the removal were analyzed using ANOVA, with the treatment, nulliparous or multiparous and baseline BS (≤2, 3≤) as fixed effects. Success of cervical ripening within 3, 6, 9 and 12 h of vaginal insert was presented as a response variable within 3, 6, 9 and 12 h.

Sample size was based on the null and alternative hypotheses that the true success rate of cervical ripening at 12 h from vaginal insert administration for subjects treated with PROPESS and placebo were (20%, 20%) and (50%, 20%), respectively, with a type one error (α) of 5%; thus, at least 52 subjects needed to be randomized to each treatment arm, namely, PROPESS and placebo (a total sample size of 104 subjects), to detect a treatment difference (Δ) of 30% with a 90% power (β) using a two‐sided test. Taking into account a withdrawal rate of approximately 10%, a total of 116 subjects was required for randomization in a 1:1 ratio to either the PROPESS or placebo arm (58 subjects to each treatment arm). The randomization was stratified by parity (nulliparous and multiparous) and by BS (BS ≤ 2 and 3≤).

## Results

### Study population

In the present study, a total of 114 subjects were randomized: 57 to the PROPESS group and 57 to the placebo group. All subjects completed the study. One subject in the placebo group with nonreassuring fetal heart rate pattern prior to administration did not receive placebo and was therefore excluded from the FAS owing to lack of efficacy evaluation.

The demographics and characteristics of the subjects are shown in Table 1, obstetric status is shown in Table 2, and BS assessment at baseline is shown in Table 3. Demographic and other baseline characteristics were generally similar between the PROPESS and placebo groups. In addition, no differences in any demographic and other baseline characteristics between nulliparous and multiparous subjects were observed in either treatment group.

**Table 1:** Demographic and baseline characteristics (full analysis set)

	PROPESS (N = 57)	Placebo (N = 56)	Total (N = 113)
Age (years)			
Mean (SD)	31.5 (5.36)	32.6 (5.62)	32.0 (5.49)
Ethnicity, n (%)			
Not Hispanic or Latino	57	56	113
Race, n (%)			
Asian	57	56	113
Baseline weight (kg)			
Mean (SD)	68.6 (14.76)	69.4 (10.79)	69.0 (12.90)
Baseline BMI (kg/m^2^)			
Mean (SD)	26.92 (5.518)	27.54 (4.031)	27.23 (4.827)

N = number of subjects.

n = number of subjects with observation.

% = percentage of subjects with observation.

There were no significant differences in patient's background between PROPESS and placebo groups.

**Table 2:** Obstetric status (full analysis set)

	PROPESS (N = 57)	Placebo (N = 56)	Total (N = 113)
Parity, n (%)			
0	47 (82.5)	44 (78.6)	91 (80.5)
1	7 (12.3)	10 (17.9)	17 (15.0)
2	2 (3.5)	2 (3.6)	4 (3.5)
3	1 (1.8)		1 (0.9)
Parity category, n (%)			
Nulliparous	47 (82.5)	44 (78.6)	91 (80.5)
Multiparous	10 (17.5)	12 (21.4)	22 (19.5)
Primary reason for induction, n (%)			
Prevention of post‐term delivery	57 (100.0)	5 (100.0)	113 (100.0)
Estimated gestational age (days)			
Mean (SD)	288.4 (1.73)	288.7 (1.51)	288.6 (1.63)
Median (P25;P75)	288.0 (287.0;289.0)	288.0 (288.0;290.0)	288.0 (288.0;290.0)
Min; max	281;292	287;292	281;292
Membrane status during first hospitalization, n (%)			
Ruptured after study drug administration	52 (91.2)	52 (92.9)	104 (92.0)
Artificial	18 (34.6)	16 (30.8)	34 (32.7)
Spontaneous	34 (65.4)	36 (69.2)	70 (67.3)
Missing		1 (1.8)	1 (0.9)

N = number of subjects.

n = number of subjects with observation.

% = percentage of subjects with observation.

There were no significant differences in patient's background between PROPESS and placebo groups.

**Table 3:** Bishop score assessment at baseline (full analysis set)

	PROPESS (N = 57)	Placebo (N = 56)	Total (N = 113)
Total BS			
Mean (SD)	2.4 (1.11)	2.3 (1.27)	2.3 (1.19)
Total BS, n (%)			
0	5 (8.8)	6 (10.7)	11 (9.7)
1	5 (8.8)	11 (19.6)	16 (14.2)
2	20 (35.1)	12 (21.4)	32 (28.3)
3	19 (33.3)	17 (30.4)	36 (31.9)
4	8 (14.0)	10 (17.9)	18 (15.9)
Bishop score category, n (%)			
0–2	30 (52.6)	29 (51.8)	59 (52.2)
3–4	27 (47.4)	27 (48.2)	54 (47.8)

N = number of subjects.

n = number of subjects with observation.

% = percentage of subjects with observation.

There were no significant differences in patients' background between PROPESS and placebo groups.

### Efficacy

#### *Cervical ripening success*

The proportion of subjects with successful cervical ripening within 12 h of IMP administration was 47.4% in the PROPESS group and 14.3% in the placebo group, and the difference was significant (treatment contrast [TC] 33.1%, 95%CI [15.6;50.0], *P* = 0.0002) as shown in Table 4.

**Table 4:** Summary of efficacy (full analysis set)

Efficacy end‐points	Treatment	n	Adjusted proportion (95% CI)	Treatment contrast versus placebo (95% CI)	*P*‐value
Proportion of subjects with cervical ripening success within 12 h of IMP administration	PROPESS	57	47.4 (34.0; 61.0)	33.1 (15.6; 50.0)	0.0002
	Placebo	56	14.3 (6.4; 26.2)		
Proportion of nulliparous subjects with cervical ripening success within 12 h of IMP administration	PROPESS	47	44.7 (30.2; 59.9)	31.0 (10.4; 49.3)	0.0014
	Placebo	44	13.6 (5.2; 27.4)		
Proportion of multiparous subjects with cervical ripening success within 12 h of IMP administration ^†^	PROPESS	10	60.0 (26.2; 87.8)	43.3 (−0.5; 75.9)	0.0743
	Placebo	12	16.7 (0.0; 6.4)		
Proportion of subjects with vaginal delivery within 12 h of IMP administration	PROPESS	57	24.6 (14.1; 37.8)	24.6 (6.5; 41.9)	<0.0001
	Placebo	56	0.0 (0.0; 6.4)		
Proportion of subjects with vaginal delivery within the first admission to hospital	PROPESS	57	81.6 (68.6; 94.7)	−2.9 (−18.6; 12.7)	0.7114
	Placebo	56	84.6 (72.1; 97.0)		
Proportion of subjects with increased BS by ≥3 points from baseline within 12 h of IMP administration	PROPESS	57	73.7 (60.3; 84.5)	43.3 (25.1; 59.2)	<0.0001
	Placebo	56	30.4 (18.8; 44.1)		
Proportion of subjects with caesarean delivery within the first admission to hospital	PROPESS	57	18.7 (5.8; 31.5)	4.7 (−10.8; 20.2)	0.5477
	Placebo	56	13.9 (1.6; 26.3)		
Proportion of subjects who received predelivery uterotonic drugs after removal of IMP	PROPESS	57	49.8 (37.5; 62.2)	−37.3 (−52.5; −22.1)	<0.0001
	Placebo	56	87.1 (75.0; 99.2)		
Proportion of subjects who underwent mechanical cervical ripening after removal of IMP	PROPESS	57	24.1 (11.0; 37.2)	−47.1 (−63.2; −30.9)	<0.0001
	Placebo	56	71.1 (58.3; 84.0)		
Proportion of subjects with BS ≥7 at onset of labor among those having onset of labor while IMP was *in‐situ*	PROPESS	36	40.8 (17.7; 63.9)	−67.0 (−100.0; 39.0)	0.2071
	Placebo	1	100.0 (0.7; 100.0)		
Duration of mechanical cervical ripening for subjects who underwent mechanical cervical ripening after removal of IMP (h) ^‡^	PROPESS	14	6.68 (2.79; 10.57)	−5.22 (−9.41; −1.04)	0.0154
	Placebo	40	11.90 (9.40; 14.40)		

† As per statistical analysis plan, a univariate analysis using normal approximation was used because the multivariate analysis model did not converge.

‡ Adjusted mean.

N, number of subjects.

PROPESS also led to a greater proportion of nulliparous subjects with cervical ripening success within 12 h of administration, compared with placebo, and the difference was significant (44.7% vs 13.6%, *P* = 0.0014). The proportion of multiparous subjects with cervical ripening success in the PROPESS group was not significantly different from that in the placebo group.

#### *Change in BS*

The proportion of subjects with increased BS by ≥3 points from baseline within 12 h was significantly higher in the PROPESS group (73.7%) compared with the placebo group (30.4%), (TC 43.3%, 95%CI [25.1;59.2], *P* < 0.0001).

#### *Proportion with vaginal delivery*

The proportion of subjects with vaginal delivery within 12 h was significantly higher in the PROPESS group (24.6%) compared with 0.0% in the placebo group (TC 24.6%, 95%CI [6.5;41.9], *P* < 0.0001).

#### *Proportion with cesarean section*

The proportion of subjects who underwent cesarean section during the first admission to hospital was 18.7% in the PROPESS group and 13.9% in the placebo group, with no significant difference observed between the two groups (*P* = 0.5477).

Cesarean section was performed because of AE: nonreassuring fetal heart rate pattern (seven subjects in the PROPESS group and three subjects in the placebo group), obstructed labor (two and four subjects, respectively), arrested labor (three and four subjects, respectively), failed induction of labor (one subject in the PROPESS group) and amniotic cavity infection (one subject in the PROPESS group). These AE were judged by the investigator to be unrelated to IMP, except for that of one subject who received PROPESS and presented nonreassuring fetal heart rate pattern.

#### *Proportion after mechanical cervical ripening procedure*

The proportion of subjects who underwent the mechanical cervical ripening procedure after IMP removal was significantly lower in the PROPESS group (24.1%) compared with 71.1% in the placebo group (TC ‐47.1%, 95% CI [−63.2; −30.9], *P* < 0.0001).

#### *Use of uterotonic drugs*

The proportion of subjects who received predelivery uterotonic drugs after removal of the IMP in the FAS was significantly lower in the PROPESS group (49.8%) compared with 87.1% in the placebo group (TC ‐37.3%, 95% CI [−52.5; −22.1], *P* < 0.0001). The dose (adjusted mean) of oxytocin was 2.52 U in the PROPESS group and 4.90 U in the placebo group, with a significantly lower dose in the PROPESS group (TC ‐2.37 U, 95% CI [−4.58; −0.17], *P* = 0.0355).

#### *Time to event*

**Figure 1:**
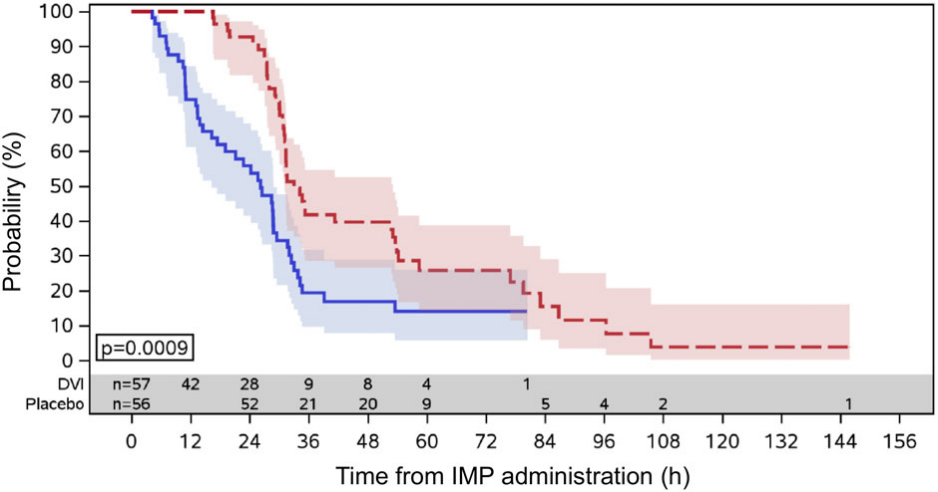
Cervical ripening success rates within 12 h (full analysis set). Plot of the survival function for the time from IMP administration to vaginal delivery within the first admission to hospital. (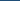) PROPESS (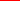) Placebo

The median time from IMP administration to vaginal delivery within the first admission to hospital was significantly lower in the PROPESS group (26.18 h) compared with 33.02 h in the placebo group in Figure 1. The time was significantly shorter in the PROPESS group compared with in the placebo based on the Cox proportional hazard model (hazard ratio: 2.51, 95% CI [1.60; 3.92], *P* < 0.0001). In addition, 75% of the subjects had vaginal delivery within 33.80 h in the PROPESS group and within 76.93 h in the placebo group.

### Safety and tolerability

Safety assessment results are summarized in Table 5.

**Table 5:** Summary of safety (safety analysis set)

	PROPESS (N = 57)		Placebo (N = 56)	
	n	(%)	n	(%)
AE (study period)	53	(93.0)	52	(92.9)
Treatment‐emergent AE (administration period)	6	(10.5)	0	
AE (neonate)	21	(36.8)	15	(26.8)
Related AE (study period)	5	(8.8)	0	
Related treatment‐emergent AE (administration period)	4	(7.0)	0	
Related AE (neonate)	1	(1.8)	0	
Severe AE (study period)	11	(19.3)	8	(14.3)
Severe AE (administration period)	1	(1.8)	0	
Severe AE (neonate)	1	(1.8)	1	(1.8)
Serious AE (study period)	22	(38.6)	18	(32.1)
Serious treatment‐emergent AE (administration period)	2	(3.5)	0	
Serious AE (neonate)	10	(17.5)	5	(8.9)
Uterine hyperstimulation	0		0	
Nonreassuring fetal heart rate pattern	13	(22.8)	12	(21.4)
Abnormal labor affecting fetus	0		1	(1.8)
Death	0		0	
AE leading to discontinuation of study	0		0	
AE leading to IMP removal	1	(1.8)	0	

N: number of subjects, n: number of subjects developing the event, %: percent of subjects. In this study, an AE was defined as described below:

A pretreatment AE: AE with onset date and time before the date and time of IMP insertion. A treatment‐emergent AE: AE with onset date and time after IMP insertion and within the time of residual drug effect or a pretreatment AE or preexisting medical condition that worsened in intensity after IMP insertion and within the time of residual drug effect, which was estimated to be approximately 30 min from the time of IMP removal. A post‐treatment intrapartum AE: AE with onset date and time after the time of IMP removal plus 30 min (i.e. plus the estimated time of residual drug effect) and not after the date and time of delivery or an existing AE or a preexisting medical condition that worsened in intensity after the date and time of IMP removal plus 30 min and not after the date and time of delivery. A postpartum AE: AE with onset date and time after the date and time of delivery or an existing AE or a preexisting medical condition that worsened in intensity after delivery and until the end of study date and time. A neonatal AE: AE in a neonate with onset date and time after the date and time of delivery and until the end of study date and time. Safety assessments of fetuses were included in the safety evaluations of subjects (mother) before delivery.

Total AE were reported to be 10.5% (six subjects) in the PROPESS group, and no subjects experienced an AE in the placebo group. Most AE were of mild or moderate severity.

Drug‐related treatment‐emergent AE were reported to be 7.0% (four subjects) in the PROPESS group: fetal heart rate deceleration abnormality, nonreassuring fetal heart rate pattern, abdominal distension, gestational hypertension and abnormal uterine contraction (1.8%, each). All subjects and neonates were alive at discharge.

Most AE that occurred during the study were those that commonly accompany labor and delivery. No new safety concerns were identified. PROPESS was well tolerated and had an acceptable safety profile in Japanese pregnant women requiring cervical ripening for prevention of post‐term delivery.

## Discussion

The present trial showed the efficacy of PROPESS against placebo for pregnant Japanese women requiring cervical ripening before labor induction for preventing post‐term pregnancy.

In this trial, the proportion of subjects with cervical ripening success within 12 h, as a primary end‐point, was significantly higher in the PROPESS group than in the placebo group (47.4% vs 14.3%, *P* = 0.0002). The results for most of the key secondary end‐points were consistent with the primary end‐point; all showed highly significant differences in favor of PROPESS. PROPESS treatment demonstrated effectiveness and safety in pregnant Japanese women requiring cervical ripening at 41 weeks of gestation compared with placebo. PROPESS will be one of the options for cervical ripening. A previous phase 1 study conducted by Ferring Pharmaceutical in Japan found that the plasma concentrations, time profile and PK parameters of PGE2 and PGE metabolites were similar between the Japanese population and non‐Japanese population administered PROPESS for 12 h. We obtained these data from Ferring Pharmaceutical to prove no difference in PK parameters between race, and the data are shown in Supplement 1.

Globally accepted guidelines recommend dinoprostone intravaginal insert for cervical ripening and inducing labor. The ACOG guidelines^2^ recommend dinoprostone use at Level A, which is described as 'Prostaglandin E analogues are effective for cervical ripening and inducing labor' and 'Intravaginal PGE2 for induction of labor in women with premature rupture of membranes appears to be safe and effective'. The National Institute for Health and Care Excellence guidelines^4^ recommend dinoprostone as the first choice in the following case: 'If a woman has preterm prelabor rupture of membranes after 34 weeks, the maternity team should discuss the following factors with her before a decision is made about whether to induce labor, using vaginal prostaglandin E2'.

In Japan, an approved PGE2 vaginal agent with high efficacy, safety and convenience has been expected by patients and physicians in the clinical setting. Mechanical cervical ripening methods are more commonly used because few options are available in the pharmacological treatment of cervical ripening and labor induction. There are some reports that compared PROPESS with mechanical methods for cervical ripening.^12^, ^13^, ^14^ One trial of 397 women with insufficient cervix ripening compared dinoprostone vaginal insert (maximum 24 h) with a transcervical in‐dwelling Foley catheter for 12 or 24 h. The proportion of women who gave vaginal delivery within 24 h was similar between the Foley catheter and dinoprostone groups.^12^

In general, guidelines recommend the use of a prostaglandin medication for cervical ripening over the use of a mechanical method. A potential disadvantage of some cervical ripening mechanical methods is that their application may be more difficult or technically challenging than that of pharmacological agents.^15^ There have been concerns that the insertion of a mechanical device into the uterus is likely to increase the risk of infection by frequent vaginal manipulations, and it was reported that the mechanical methods increased the risk of infection compared with pharmacological agents .^16^ In addition, the use of balloons for cervical ripening may be associated with the risk of umbilical cord prolapse.^17^

Mechanical cervical ripening may be associated with pain as the cervix is physically dilated. A report showed that the need for analgesia tends to be higher in women who received a balloon catheter than in those who received intravaginal dinoprostone.^18^

In this trial, the safety assessment results of PROPESS were consistent with the safety profile known in labor and delivery settings, as well as the findings from previous trials and other dinoprostone formulations. No new safety concerns and issues were identified. PROPESS was thus considered well‐tolerated and had an acceptable safety profile, as determined by the following reasons. During the trial, no subjects and neonates died. Most reported AE were classified as being of either mild or moderate intensity. The overall incidence of AE in the PROPESS group (93.0%) was similar to that in the placebo group (92.9%) during the trial. The most frequent AE was after birth pain (56.1% and 55.4%, respectively), followed by anemia (36.8% and 39.3%, respectively). The reported AE are known to be common in labor and delivery settings. No unexpected or clinically concerning AE were observed during the trial.

PROPESS, as a cervical ripening drug for pregnant women, is considered safer than other administration routes and formulations (e.g. vaginal gel, oral tablet and intravenous bolus injection) due to the following reasons: (i) it allows easy and quick retrieval after insertion when required, and immediate removal is possible if any adverse reaction should occur; (ii) it is the only product with controlled and consistent release of PGE2 at a rate of approximately 0.3 mg/h over 12 h. Among these features, the main advantage of PROPESS over other dinoprostone formulations is its easy removal and easy termination of dosing at the onset of labor or in the event of uterine hyperstimulation or other adverse events.^19^ In case of insufficient cervical ripening, compared with mechanical dilation, PROPESS significantly reduces oxytocin administration and time to delivery using analgesia, as well as improves BS and maternal satisfaction.^14^, ^20^, ^21^, ^22^

A limitation of this study was not investigated in pregnant women at term <41 weeks 0 day and > 42 weeks 0 day of gestation. It is important to investigate this population, so we would like to investigate the efficacy and safety in pregnant women who have crossed the maximum number of gestational weeks. The number of nulliparous women was 80% and multiparous women was 20%; this is not intended but casual. The cervical ripening rate was higher in multiparous women; thus, it may affect the total success rate.

## Conclusion

PROPESS administration for a maximum of 12 h was an effective and well‐tolerated treatment for pregnant Japanese women requiring cervical ripening (at 41 weeks of gestation). Hence, PROPESS could be considered a treatment option for cervical ripening.

## Disclosure

This study was funded by Ferring Pharmaceutical. Hiroaki Itoh, Keisuke Ishii, Naoya Shigeta, Atsuo Itakura, Hiromi Hamada, Takeshi Nagamatsu and Tomohiko Ishida have no conflict of interest. Yasuaki Bungyoku, Ali Falahati, Miori Tomisaka and Mikiya Kitamura are employees of Ferring Pharmaceuticals Co. Ltd.

## Supporting information


**Figure S1** Pharmacokinetic study conducted on nonpregnant women
