# Supplementary figures and images for: Efficacy and safety of controlled‐release dinoprostone vaginal delivery system (PROPESS) in Japanese pregnant women requiring cervical ripening: Results from a multicenter, randomized, double‐blind, placebo‐controlled phase III study
Source: J Obstet Gynaecol Res. 2020 Oct 22;47(1):216–25. doi: 10.1111/jog.14472 (PMC7820955; doi:10.1111/jog.14472)

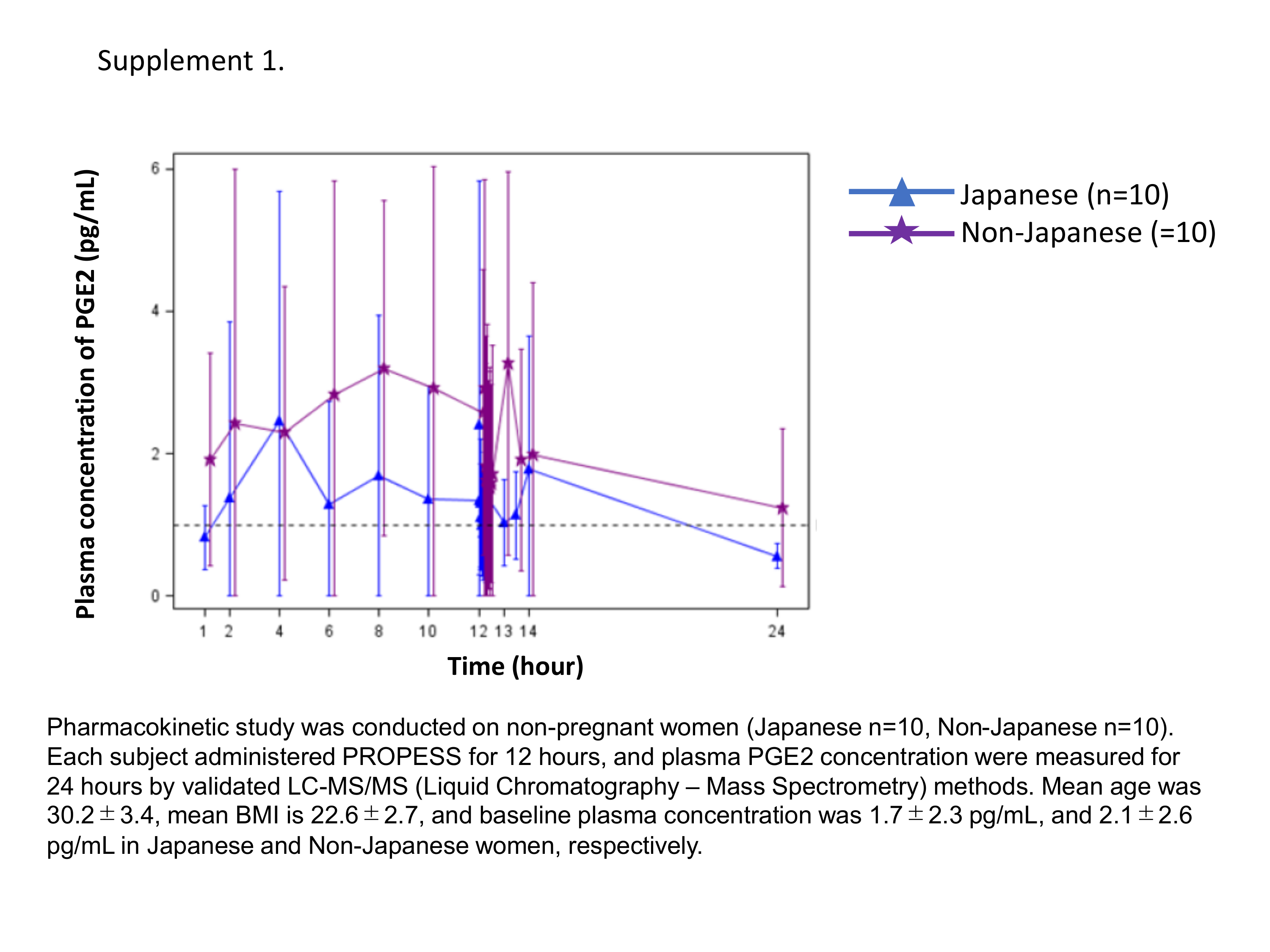

Supplement: Supplementary file 1 — Figure S1 Pharmacokinetic study conducted on nonpregnant women [file JOG-47-216-s001.tif]
